# Prevalence of hypercalcemia among cancer patients in the United States

**DOI:** 10.1002/cam4.749

**Published:** 2016-06-05

**Authors:** Victor M. Gastanaga, Lee S. Schwartzberg, Rajul K. Jain, Melissa Pirolli, David Quach, Jane M. Quigley, George Mu, W. Scott Stryker, Alexander Liede

**Affiliations:** ^1^ Amgen Inc.Thousand Oaks and South San FranciscoCalifornia; ^2^ The West ClinicMemphisTennessee; ^3^ Kite Pharma Inc.Santa MonicaCalifornia; ^4^ IMS HealthPlymouth MeetingPennsylvania; ^5^ PRA Health SciencesBlue BellPennsylvania; ^6^ Glaxo Smith KlineCollegevillePennsylvania

**Keywords:** Electronic health records, hypercalcemia, hypercalcemia of malignancy, prevalence

## Abstract

Hypercalcemia of malignancy (HCM) is a serious metabolic complication whose population‐based prevalence has not been quantified. Rates of HCM differ by tumor type, with highest rates reported in multiple myeloma and lowest among colorectal and prostate cancer patients. This analysis estimates HCM prevalence in the US. This retrospective study used the Oncology Services Comprehensive Electronic Records (OSCER) warehouse of electronic health records (EHR) including laboratory values from 569000 patients treated at 565 oncology outpatient sites. OSCER data were projected to the national level by linking EHR to claims data. Cancer patients included were ≥18 years, and had serum calcium (Ca) and albumin (for corrected serum Ca [CSC]) records. Period prevalence was estimated by HCM CTCAE grade, tumor type, and year (2009–2013). Estimates were adjusted to capture patients diagnosed with HCM outside oncology practices based on a subset of patients linkable to office and hospital data. The analysis included 68023 (2009) to 121482 (2013) cancer patients. In 2013, patients with HCM had a median of six Ca tests, 69.7% had chemotherapy, and 34% received bone modifying agents. HCM rates were highest for multiple myeloma patients (7.5% [2012]–10.2% [2010]), lowest for prostate cancer (1.4% [2012]–2.1% [2011]).The estimated adjusted annual prevalence of HCM from 2009 to 2013 was 95441, 96281, 89797, 70158, and 71744, respectively. HCM affected 2.0–2.8% of all cancer patients. EHR data from oncology clinics were critical for this study because these data contain results from laboratory studies (i.e., serum calcium values) that are routinely ordered in that setting. We estimated that the prevalence of HCM in the US in 2013 is 71744, affecting approximately 2% of cancer patients overall. This percentage differs by tumor type and appears to have decreased over the five‐year study period.

## Introduction

Hypercalcemia is defined by serum calcium level greater than the upper limit of the normal (ULN) reference range of 10.5 mg/dL or 2.5 mmol/L 1, and is associated with two main causes: (1) tumor‐induced hypercalcemia or hypercalcemia of malignancy (HCM), and (2) primary hyperparathyroidism. Malignancy is the most frequent cause of hypercalcemia in a hospital patient population 1, 2, 3, 4, whereas primary hyperparathyroidism is a more common cause of elevated blood calcium in the general population 2.

We define HCM as hypercalcemia observed in cancer patients, regardless of etiology. HCM can be observed in any cancer type 5. Although almost one‐third of HCM cases are not due to cancer 4, the pathophysiological mechanism in the remaining two‐thirds is variable depending on the primary cancer. Regardless of mechanism, HCM generally occurs late in the course of malignancy 6.

General and neurological status of the patient as well as the velocity of increase in calcium levels may influence symptom severity which may sometimes appear disproportionate according to the actual levels of serum calcium 7. Symptoms of HCM are not specific and may involve renal (polyuria, dehydration, renal failure), gastrointestinal (polydipsia, anorexia, nausea and/or vomit, constipation), central nervous (fatigue, confusion, delirium, cognitive impairment, depression, ataxia, muscle weakness, psychotic attitudes, coma), and cardiovascular systems (hypertension, bradycardia, electrocardiogram (ECG) alterations, and orthostatic hypotension) 6, 7, although symptom severity has been correlated with serum calcium levels 8, 9.

Hypercalcemia is one of the most common metabolic complications of malignancy, but its incidence and prevalence have not been quantified systematically. It has been estimated from clinical trials or retrospective case series that between 3% and 30% of cancer patients experience hypercalcemia at some point, and that rates of HCM differ by primary tumor diagnosis 2, 5, 7, 10. Conversely, HCM occurs much less frequently in pediatric malignancies, with estimates ranging from 0.4% to 1.3% 11, 12, 13, occurring in all hematological and solid tumor types 11.

In the US and Europe, primary tumors of the lung and breast 2, 5, 6, and multiple myeloma 10 are the most common hypercalcemia‐associated malignancies, followed by squamous‐cell carcinoma of the head and neck (SCCHN), renal, and ovarian cancer. HCM has been associated with squamous histology, and was rarely found in patients with colorectal and prostate cancers 2, 10. Lung cancer, breast cancer, and multiple myeloma account for >50% of all HCM cases among patients diagnosed with cancer.

Patients with HCM often present with renal insufficiency, which is multifactorial in etiology. A first step of therapy is usually to restore proper fluid balance as dehydration is commonly encountered. Enhanced bone resorption represents the main cause of HCM and thus, the anti‐bone resorptive therapies also represent an appropriate therapeutic approach when hydration alone is ineffective in normalizing calcium values. Bisphosphonates (pamidronate and zoledronic acid) represent the mainstay of treatment. Denosumab, a fully human monoclonal antibody against RANKL, may offer a new treatment option for HCM as demonstrated in a recent study 14 with complete response over the course of the study among 64% of patients with persistent or relapsed HCM despite recent bisphosphonate treatment.

We estimated the prevalence of HCM by grade and tumor type using electronic health records (EHR) from oncology practices across the United States. The widespread adoption of EHR by community oncology practices makes this a valuable tool for observational studies in oncology. Specifically, EHR captures routine laboratory results (i.e., serum calcium and albumin values). We examined HCM trends over a recent time period (2009–2013) including the use of bone resorptive therapies (intravenous bisphosphonates [pamidronate and zoledronic acid] and denosumab). We also described renal impairment among patients with HCM, and survival for a subset of patients with vital status via external data linkage.

## Methods

This study was conducted with EHR data using the Oncology Services Comprehensive Electronic Records (OSCER) database. OSCER includes outpatient data for a representative sample of more than 569,000 cancer patients treated at 52 community and hospital‐affiliated oncology practices (565 clinics) from 2004 forward. Patients reside in all 50 states and all payer types were represented (commercial, Medicare, Medicaid, self‐pay, and other). Patient records in OSCER were de‐identified and fully compliant with the Health Insurance Portability and Accountability Act (HIPAA) of 1996.

During each oncology clinic visit, detailed data including ICD‐9‐CM (International Classification of Diseases Classification 9th Revision Clinical Modification) diagnosis codes, CPT‐4 (Current Procedural Terminology) procedure codes, laboratory test results, and treatments administered or prescribed were captured, along with the relevant service dates, in the EHR. Laboratory test dates, results, applicable units and normal reference ranges were typically entered directly into the EHR.

We identified cancer patients with at least two visits and at least one serum calcium value between 1 January 2009 and 31 December 2013. Cancer patients were selected from OSCER practices that reported regularly during the study period, and were required to have known gender, year of birth, and be at least 18 years old at the time of cancer diagnosis. Patients who received investigational agents were excluded.

We estimated the annual prevalence of hypercalcemia, defined as the number of all eligible cancer patients who have HCM over a year. Annual prevalence can be expressed as a percentage, as follows:

$$\text{Annual prevalence ratio}\;=\;\frac{\text{Number of HCM cases that occurred in a year}}{\text{Number of eligible cancer patients in the same period}.}$$

Serum calcium is reported in mg/dL. Hypercalcemia was defined according to corrected serum calcium (CSC) levels by Common Terminology Criteria for Adverse Events (CTCAE) grade 15:

- Grade 1: CSC between ULN and 11.5 mg/dL
- Grade 2: CSC between >11.5–12.5
- Grade 3: CSC between >12.5–13.5
- Grade 4: CSC >13.5

Note: CSC = 0.8 ×  (4 – serum albumin) + serum calcium 16, 17. For consistency with clinical trial definitions of HCM and complete response 14, we also analyzed grade ≥1 using the CSC >10.8 mg/dL threshold.

In order to project OSCER prevalence to the US national level, we estimated relationships between key data elements utilizing patient EHR linked to prescription and medical office claims data. Prescription and medical office claims were projected by tumor type. The claims totals were then used to create factors to project the EHR sample to the US population 18. These factors were used to project patients by tumor type with laboratory values that are nationally representative of treated cancer patients. Estimates were adjusted to capture patients diagnosed with HCM outside oncology practices using a subset of patients linkable to office and hospital data. Prevalence estimates are reported overall and by tumor type: lung, breast, colorectal, prostate, renal, multiple myeloma, and all other cancers.

The beginning of follow‐up occurred on the patient's index date (first office visit recorded on or after 1 January 2009 among patients with a prior cancer diagnosis), and continued until end of follow‐up in the database.

The demographic and clinical characteristics of the study population were summarized. We estimated the prevalence of HCM from 2009 to 2013 for the overall study population, and stratified by tumor type, metastasis (yes/no), and type of metastasis (visceral vs. other). We also examined distribution of these patients across eGFR categories: <30, 30–59, 60–89, and ≥90 mL/min/1.73 m^2^. We determined the distribution of patients using bone‐targeted agents (BTA) denosumab, zoledronic acid, and pamidronate by HCM grade and tumor type.

In exploratory analyses, we assessed differences in survival between hypercalcemic and normocalcemic patients using Kaplan–Meier curves, including the log‐rank test. A Cox proportional hazards regression model with time since diagnosis as the time scale, with hypercalcemia as a time‐varying covariate (defined as 1 for months with elevated calcium and 0 otherwise) was used to generate hazard ratios (HRs) with 95% confidence intervals. The Cox regression proportional hazards assumption was validated using a chi‐squared test. Statistical tests were two‐sided and a *P* < 0.05 was considered statistically significant. Statistical analyses were performed using SAS^®^ software for Windows, version 9.3 (SAS Institute Inc., Cary, NC).

## Results

**Figure 1:**
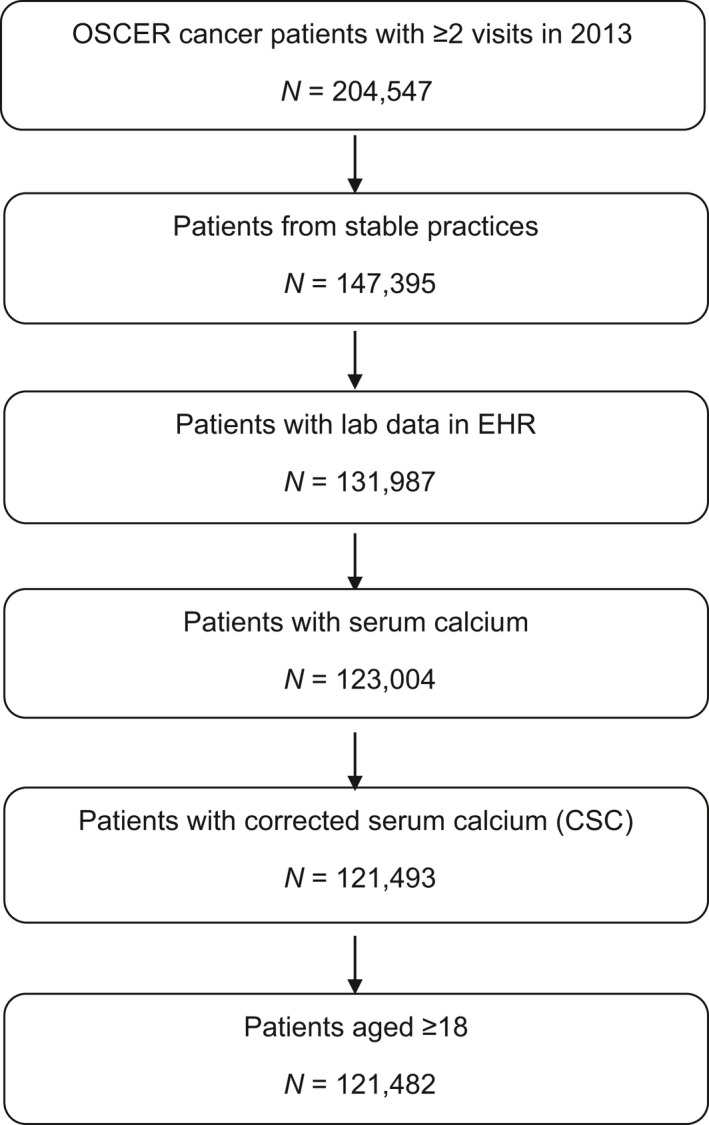
Cohort identification (2013 shown).

The analysis included 68023 patients in 2009 to 121482 patients in 2013 (Fig. 1), projected to 3.4 to 3.6 million cancer patients nationwide. Females comprised 63% of the study population, and 58% of HCM patients (Table 1). Sixty percent of cancer patients and 65% of HCM patients in the study cohort were 65 years of age or older. Among all HCM patients, 45% had metastases versus 22% among all cancer patients. Visceral metastases were present in 8.9% of HCM and 4.8% of all cancer patients. While 32% of HCM patients received BTA treatment any time after cancer diagnosis during the study period, only 8% of normocalcemic patients received it. The fraction of HCM patients who received BTA after their first elevated CSC value was 28%.

**Table 1:** Cancer patients in the OSCER database

	Cancer patients in OSCER (2009–2013)^a^	
	All patients	HCM^b^ patients
OSCER cancer patients^a^ (Raw Data)	224,817	7388
Median annual no. of Ca tests	6	11
Median days from cancer Dx to HCM	175	155
US patients with HCM (Projected)	8,281,297	344,411
Age 65 or older	60%	65%
Female	63%	58%
Lung cancer	27,618	1605
Breast cancer	73,458	1620
Colorectal cancer	23,574	560
Prostate cancer	8527	173
Renal cancer	2661	207
Other solid tumor	45,115	1571
Multiple myeloma	6328	736
Non‐hodgkin lymphoma	17,786	596
Other hematologic	19,750	321
% Metastatic (solid tumors only)	22.1%	45.2%
% Visceral metastases only	3.5%	5.0%
% Visceral and bone metastases	1.3%	3.9%
Receiving chemotherapy	44.8%	69.7%
Receiving bone targeting agents^c^	8.3%	31.4%
% of patients receiving BTAs by grade		
Grade 0 (No HCM)	7.3%	N.A.
Grade 1	0.5%	14.3%
Grade 2	0.3%	8.7%
Grade 3	0.2%	4.6%
Grade 4	0.1%	3.7%

OSCER, oncology services comprehensive electronic records; HCM, hypercalcemia of malignancy.

a Unique patients. Values assessed for the year the patient first meets the study criteria or first records high calcium.

b HCM defined as CTCAE grade ≥1.

c Denosumab, zoledronic acid, pamidronate.

Demographic and clinical characteristics of HCM patients, by year, are shown in Table 2. In 2013, patients with confirmed HCM had a median of six Ca tests, 70% received chemotherapy, and 34% had bone modifying agents. The overall percentage of patients with metastases, receiving chemotherapy, and receiving BTA were stable over the 5‐year study period. However, BTA use among patients in the higher HCM grades has increased in 2012–2013 relative to 2009–2011.

**Table 2:** Cancer patients with HCM in the OSCER database

	HCM patients				
	2009	2010	2011	2012	2013
OSCER patients with HCM^a^ (Raw data)	1584	1697	1866	1563	1750
Median annual no. of Ca tests	6	7	5	5	6
Median days from cancer Dx to HCM	158	232	231	252	249
US patients with HCM (Projected)	95,441	96,281	89,797	70,158	71,744
Age 65 or older	59%	62%	61%	66%	68%
Female	58%	59%	59%	61%	60%
Lung cancer	377	368	370	315	326
Breast cancer	361	381	424	394	412
Colorectal cancer	143	157	148	94	111
Prostate cancer	32	30	53	37	51
Renal cancer	37	54	55	42	39
Other solid tumor	324	359	390	297	348
Multiple myeloma	142	160	191	172	218
Non‐Hodgkin lymphoma	109	118	147	138	159
Other hematologic	59	70	88	74	86
% Metastatic (solid tumors only)	47.4%	43.7%	46.3%	43.3%	44.0%
% Visceral metastases only	4.5%	4.6%	4.4%	5.5%	5.7%
% Visceral and bone metastases	3.0%	2.6%	4.2%	5.3%	4.7%
% Bone metastases only	19.4%	17.5%	18.6%	16.9%	16.7%
Receiving chemotherapy	66.0%	71.2%	71.2%	70.7%	69.7%
Receiving bone targeting agents^b^	31.3%	32.2%	31.7%	34.8%	34.0%
% of patients receiving BTAs by grade					
Grade 1	16.4%	16.5%	16.2%	16.4%	16.0%
Grade 2	8.3%	8.5%	8.4%	9.1%	9.7%
Grade 3	3.7%	3.7%	3.7%	5.4%	5.3%
Grade 4	2.9%	3.6%	3.4%	3.8%	3.1%

a HCM defined as CTCAE grade ≥1.

b Denosumab, zoledronic acid, pamidronate.

Treatment rates with BTA varied by HCM status. Among all cancer patients, the average number of administrations was 9.9 (standard deviation, 11.3). Among normocalcemic cancer patients, the average was 10.0 (11.2). Among patients with HCM grade ≥1, grade ≥2, grade ≥3, and grade 4, the average number of administrations were 8.8 (12.0), 6.2 (9.2), 5.7 (8.7), and 5.5 (9.5), respectively. For HCM patients, these frequencies were assessed any time on or after the first HCM diagnosis. For normocalcemic patients, BTA frequencies were assessed on or after the first cancer diagnosis. Length of BTA administration was similar for both groups, with average (standard deviation) 13.4 (14.5) and 13.4 (14.4) months for HCM and non‐HCM patients, respectively.

BTA use following HCM diagnosis was highest among multiple myeloma (58%), prostate (48%), and renal (41%) cancer patients, and lowest among colorectal (10%), NHL (19%), and other cancers (17%). BTA use averaged 28% among hypercalcemic patients following their HCM diagnosis. BTA use averaged 28% among all cancer patients’ HCM. Among normocalcemic patients, BTA use was stable at approximately 9% over the study period. Zoledronic acid accounted for approximately three quarters (74%) of BTA use among HCM patients.

**Figure 2:**
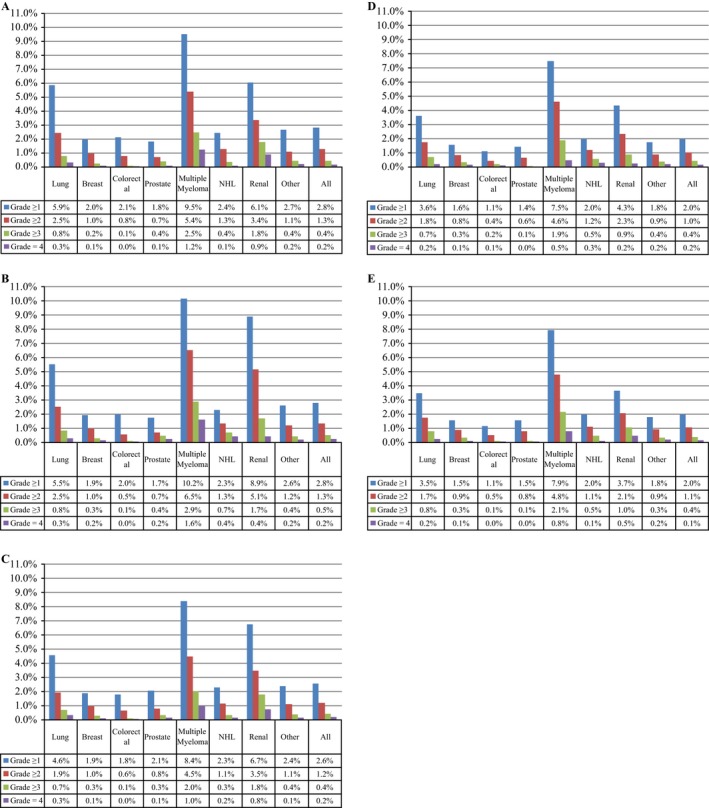
Hypercalcemia of malignancy (HCM) prevalence by grade, tumor type, and year: (A) 2009,(B) 2010. (C) 2011. (D) 2012. (E) 2013.

**Figure 3:**
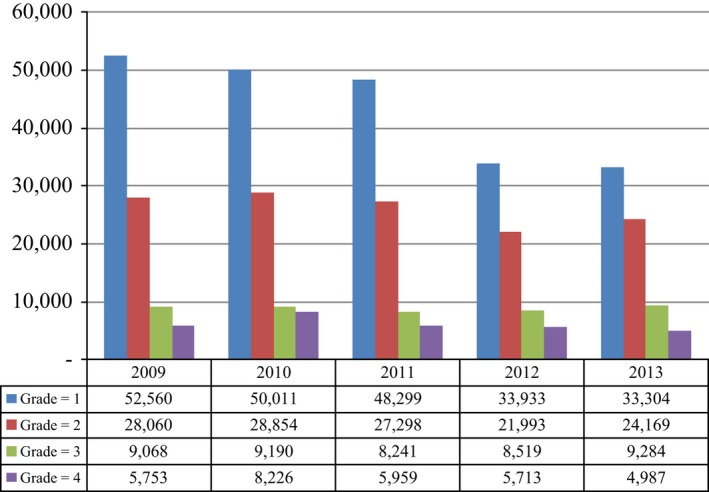
Annual prevalence of hypercalcemia of malignancy by grade and year (2009–2013).

Estimated annual (adjusted) prevalence of HCM from 2009 to 2013 in the US was 95441, 96281, 89797, 70158, and 71744, respectively. HCM is estimated to have affected 2.8% and 2.0% of cancer patients in 2009 and 2013, respectively. HCM rates were higher for multiple myeloma patients (7.5% [2012]–10.2% [2010]), lowest for prostate cancer (1.4% [2012] –2.1% [2011]). Over the 5‐year study period, we found that 3.6% of head and neck cancer patients experienced HCM, compared to 3.5% among all patients with solid tumors. The projected prevalence of HCM among cancer patients in the US by year, tumor type, and grade are shown in Figure 2 as percentages within each cancer type. Projected HCM prevalence counts at the US national level by tumor type and grade in 2013 are shown in Table 3. Finally, the projected HCM prevalence by grade over time is shown on Figure 3.

**Table 3:** HCM prevalence by tumor type in the US (2013)

		Corrected Calcium	Hypercalcemia			
			Grade 1	Grade 2	Grade 3	Grade 4
2013 patients		<10.8	10.8–11.5	>11.5–12.5	>12.5–13.5	>13.5
Tumor type	Projected count	# (%)	#% (LCL, UCL)	#% (LCL, UCL)	#% (LCL, UCL)	#% (LCL, UCL)
Total cancer patients	3,625,602	3,553,858 98.0%	33,3040.9% (0.9, 1.0)	24,1690.7% (0.6, 0.7)	92840.3% (0.2, 0.3)	49870.1% (0.0, 0.2)
Lung cancer	323,012	311,820 96.5%	55771.7% (1.5, 1.9)	30630.9% (0.8, 1.1)	17640.5% (0.4, 0.7)	7890.2% (0.0, 0.3)
Breast cancer	1,154,905	1,137,133 98.5%	77170.7% (0.6, 0.7)	64640.6% (0.5, 0.6)	26930.2% (0.2, 0.3)	8980.1% (0.0, 0.1)
Colorectal cancer	355,023	350,959 98.9%	23300.7% (0.5, 0.8)	13160.4% (0.3, 0.5)	2990.1% (0.0, 0.1)	120<0.0% (0.0, 0.1)
Prostate cancer	396,502	390,365 98.5%	30450.8% (0.5, 1.0)	27060.7% (0.4, 0.9)	193<0.0% (─, 0.1)	193<0.0% (─, 0.1)
Multiple myeloma	95,626	88,035 92.1%	30143.2% (2.7, 3.6)	25272.6% (2.2, 3.1)	12871.3% (1.0, 1.7)	7630.8% (0.0, 1.1)
NHL	300,684	294,812 98%	24970.8% (0.7, 1.0)	19690.7% (0.5, 0.8)	10780.4% (0.2, 0.5)	3280.1% (0.0, 0.2)
Renal cancer	64,603	62,232 96.3%	10261.6% (0.9, 2.2)	6721.0% (0.5, 1.5)	3730.6% (0.2, 1.0)	2990.5% (0.0, 0.8)
Other cancer	935,246	918,502 98.2%	80980.9% (0.8, 1.0)	54520.6% (0.5, 0.7)	15970.2% (0.1, 0.2)	15970.2% (0.0, 0.2)

Only 14% of all cancer patients in the study sample had a normal eGFR ≥90 mL/min/1.73 m^2^ (Table 4), while 6.6% had eGFR <30. Of normocalcemic patients, 6.1% had eGFR <30, while 14.2% had eGFR ≥90. In contrast, 19% of HCM patients (grade ≥1) had eGFR <30, while only 9% had normal eGFR ≥90.

**Table 4:** Renal function among cancer patients

Status	Number of patients with eGFR	% by eGFR measurement^a^ 2009–2013			
		<30	30–59	60–89	90+
Total cancer patients in OSCER	222,701	6.6%	34.9%	44.6%	14.0%
All HCM patients (grade ≥1)	7304	19.4%	44.1%	27.6%	8.9%
HCM grade ≥2	1819	23.6%	39.5%	25.4%	11.5%
HCM grade ≥3	579	26.6%	39.4%	23.1%	10.9%
HCM grade 4	204	33.3%	36.8%	22.1%	7.8%
No hypercalcemia Dx^b^	215,397	6.1%	34.5%	45.2%	14.2%

a Lowest eGFR at any point on or after hypercalcemia diagnosis.

b Lowest eGFR at any point on or after cancer diagnosis.

**Figure 4:**
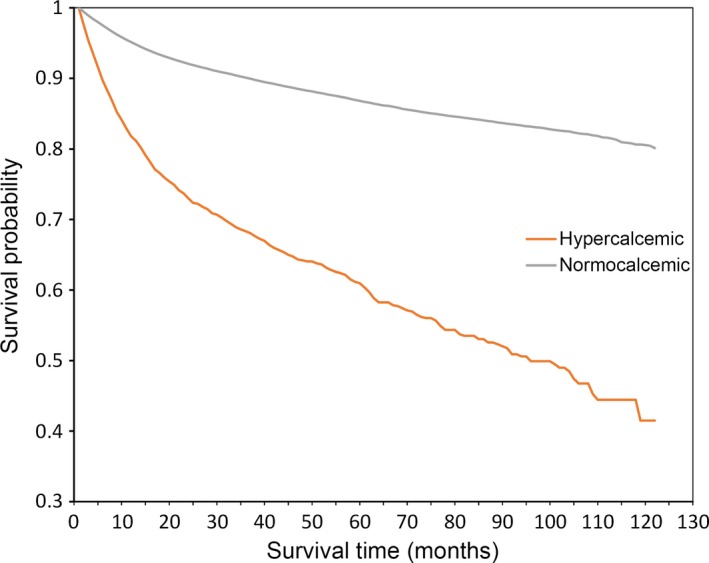
Kaplan–Meier survival curves by hypercalcemic status.

We ascertained mortality based on vital records from the Experian consumer database (Table 5). Approximately half (52%) of all OSCER cancer patients included in the analysis were linkable to this database. The linked patients had similar distribution of age, gender, tumor type and other demographics compared to the overall population. Of these linked cancer patients, 12% (13,652 of 117,344) died during the study period. The mortality rate among HCM (grade ≥1) patients was 34%, while only 11% of normocalcemic patients died (Table 5). By grade, the unadjusted risk of death was 38% for grade ≥2, and 42% for grade ≥3 and grade 4. From the time of first cancer diagnosis, normocalcemic patients had 22.5 months of average survival (median 14 months), compared to an average of 18.9 months (median 11 months) among patients with HCM of any grade. The difference in survival between the normocalcemic and hypercalcemic patients over time was statistically significant (log‐rank test: *χ* ^2^  = 1949, *P* < 0.0001) (Fig. 4). A Cox proportional hazards model adding hypercalcemia as a time‐varying covariate (defined as 1 for months with elevated calcium and 0 otherwise), cancer type, and hypercalcemia grade yielded similar results (Table 6). Compared to solid tumors not separately analyzed, breast cancer patients had approximately 64% lower risk of death. Similarly, risk of death was decreased by 23%, 58%, and 50% for colorectal, hematologic, and renal cancer patients, respectively. Compared to grade 1 hypercalcemia, risk of death was 44% and 61% higher for patients with HCM grades 3 and 4, respectively. The hazard ratio associated with elevated calcium months was 4.9 (*P* < 0.0001). Although these analyses did not account for all potentially relevant clinical variables associated with survival, these rates are consistent with a strong association between HCM (and its severity) and risk of death.

**Table 5:** Mortality by HCM status and grade among OSCER patients

	Cancer patients 2009–2013				
	Follow‐up time after cancer diagnosis				
	Frequency			Survival (months)	
	Total^a^	Deaths	%	Mean	Median
Total cancer patients *(OSCER sample)*	117,344	13,652	11.6%	22.2	14
All hypercalcemia patients^a^ (grade ≥1)	3185	1094	34.3%	18.9	11
HCM grade ≥2	731	280	38.3%	14.7	8
HCM grade ≥3	232	98	42.2%	15.5	7
HCM grade 4	95	40	42.1%	14.6	8
No hypercalcemia^b^	114,159	12,558	11.0%	22.5	14

a Total: All patients linkable to the Experian consumer database (from cancer diagnosis).

b Log‐rank test of difference in survival between hypercalcemic and normocalcemic cancer patients (*χ* ^2^  = 1949, *P *<* 0*.0001).

**Table 6:** Multivariate analysis of mortality risk

	HR	95% CI		*P*‐Value
Calcemic month				
Normal	Ref			
Elevated	4.90	4.29	5.48	<0.0001
Cancer type				
All other solid	Ref			
Breast	0.36	0.30	0.44	<0.0001
Colorectal	0.77	0.61	0.97	0.0288
Head and neck	1.11	0.81	1.51	0.5308
Hematologic	0.42	0.35	0.51	<0.0001
Lung	1.16	0.98	1.37	0.0763
Prostate	0.50	0.31	0.82	0.0061
Renal	0.82	0.56	1.18	0.2833
Hypercalcemia grade				
Grade 1	Ref			
Grade 2	1.11	0.94	1.31	0.2125
Grade 3	1.44	1.10	1.88	0.0082
Grade 4	1.61	1.17	2.22	0.0034

Only hypercalcemic patients were included in the Cox regression.

HR: hazard ratio; CI: confidence interval.

## Discussion

This study of EHR data is the first to comprehensively estimate the prevalence of HCM among cancer patients treated at oncology practices across the United States, while also reporting on the use of BTAs, renal function, and survival. Our data confirmed that HCM is more common among patients diagnosed with lung cancer, renal cancer, and multiple myeloma.

The incidence of HCM may be decreasing in the US as a direct result of earlier and prolonged use of bisphosphonates among patients with bone metastases. In a randomized controlled trial of patients with bone metastases from solid tumors other than breast or prostate cancer who had no more than a single exposure to bisphosphonates, HCM occurred in only 1% of the cases treated with zoledronic acid compared with 3% observed among the placebo group 19. We confirmed that HCM prevalence declined from 2009 to 2013, although the specific reasons for this decline were not explored. BTA use remained stable (~9%) over the 5‐year study period.

Recently, there has been great interest in real‐world data, electronic health data, and the promise of “big data” 20, and how to translate them into new knowledge 21. The adoption of EHR is particularly relevant in oncology, relative to other medical specialties, where adoption of EHR has become part of the clinical care of patients 22. The value of EHR is further evidenced by the American Society of Clinical Oncology (ASCO) launch of the CancerLinQ^™^ learning system to improve patients’ outcomes and quality of life based on EHR data 23, 24. In oncology, data such as routine laboratory results (e.g., serum calcium and albumin values) are now part of EHR. This study demonstrated the ability to characterize prevalence of a cancer‐related condition using data found in EHR.

We conclude that 2–3% of US cancer patients are affected by HCM each year. While HCM is less common in prostate cancer and breast cancer, these patients make up 34% of cancer patients with HCM. Myeloma patients have the highest prevalence, but represent only 10% of the HCM population. We found that the frequency of BTA use was inversely correlated with HCM severity, while decreased renal function and mortality were directly associated with HCM severity. Further work may be needed to evaluate these associations in the context of comorbidities and treatments among this patient population.

These real‐world data analyses of EHR from patients treated at oncology practices across the country further our understanding and the literature on HCM, where studies have been limited to single institution case series or clinical trials. A retrospective analysis of laboratory data from 7667 new cancer patients registered at a US medical center in 1989 reported an overall incidence of HCM of 1.15% 25. Across tumor types, we found that 2% of patients were affected with HCM grade 1 or higher among a sample of 121,482 cancer patients treated at oncology clinics across the US in 2013. Also, the proportion of patients diagnosed with metastases based on ICD–9 coding was twice as high among hypercalcemic patients compared to all cancer patients (44% in HCM vs. 22% overall) although the proportion of patients with metastases were much lower than described in the Vassilopoulou‐Sellin study 25 (70–75% in hypercalcemic patients vs. 37% in normocalcemic patients). It should be noted that the sensitivity of ICD‐9 coding for identification of patients with metastases is lower than 100%; recently estimated to be 98% for identifying bone metastases among breast, lung, and prostate cancer patients in OSCER 26.

It is important to note a few specific limitations in our study. Projected annual HCM prevalence varied over the last 5 years and may reflect changes in the patient and practice source data, as well as our ability to link EHR to hospitalization data to adjust for HCM occurring outside OSCER sites. While the OSCER database provides detailed clinical data for a large sample of cancer patients, these data are generally limited to services that are provided in participating oncology clinics. Therapies administered outside of these clinics would not be captured, which may result in underestimation of BTA use. In fact, we found that only 28% of HCM patients are treated with a BTA following their HCM diagnosis. We also note that patients who were diagnosed with cancer or HCM later in the study period had shorter follow‐up, which may also result in underestimation of the use of BTAs and lower rates of mortality. The survival analyses presented here were not comprehensive in accounting for relevant clinical variables associated with survival, and therefore should be interpreted as exploratory in nature. In particular, we found longer survival rates compared to prior reports 27, 28, 29.

Although oncology practices contributing data to the OSCER database are geographically spread across all states and agnostic to payer types and therefore representative of the broader cancer population in the US, inclusion of sites is inherently based on EHR implementation by clinics, and clinics may potentially differ in how laboratory tests are recorded relative to clinics that have not adopted EHR. The eligibility criteria for this study were nonrestrictive and therefore, we believe additional selection bias within the database would be minimal. Finally, we note that creatinine measurements and resulting eGFR estimates have inherent limitations and may be unreliable under certain conditions such as extreme age or body size, malnutrition or obesity, skeletal muscle disease, paraplegia or quadriplegia, vegetarian diet, and pregnancy.

Further to the promise of electronic health data and big data, these analyses were reviewed by the US Food and Drug Administration and the Australian authorities and were determined as robust epidemiologic evidence for orphan drug designation for a new treatment for patients with HCM refractory to intravenous bisphosphonates. The OSCER EHR data warehouse and the real‐world data approach have the potential to improve and expedite similar estimates in pharmacoepidemiology studies.

## Related Publications

Similar data were presented in two posters, “Prevalence of Hypercalcemia of Malignancy in the United States” at the American Society for Bone and Mineral Research Annual Meeting, Baltimore, Maryland, October 2013 30; and “Prevalence of Hypercalcemia of Malignancy in the United States: Projection Methods Using Oncology Electronic Health Records (EHR)” at the European Cancer Congress, Amsterdam, Netherlands, September 2013 31.

## Conflict of Interest

V.M.G., R.K.J., W.S.S., and A.L. are employees or former employees of Amgen. L.S.S., M.P., D.Q., J.M.Q., and G.M. have acted as consultants for Amgen.
